# Sheet and void porous media models for brain interstitial space

**DOI:** 10.1098/rsif.2023.0223

**Published:** 2023-08-09

**Authors:** Charles Nicholson

**Affiliations:** Department of Neuroscience and Physiology, New York University Grossman School of Medicine, New York, NY 10016, USA

**Keywords:** brain extracellular space, diffusion, porosity, tortuosity, dead space, sleep

## Abstract

The interstitial space (ISS) component of brain extracellular space resembles an unconsolidated porous medium. Previous analysis of the diffusion of small molecules in this domain shows that the typical porosity is 0.2 and typical tortuosity 1.6. An ensemble of cubic cells separated by uniform sheets of ISS cannot generate the measured tortuosity, even if some of the tortuosity value is attributed to interstitial viscosity, so more complex models are needed. Here two models are analysed: the corner cubic void (CCV) and the edge tunnel void (ETV). Both models incorporate dead spaces formed from local expansions of the ISS to increase geometrical tortuosity. Using Monte Carlo simulation of diffusion it is found that in the range of normal porosities, the square of the tortuosity is a linear function of the ratio of void to sheet volumes for the CCV model and this model can generate the experimentally observed tortuosities. For abnormally high porosities, however, the linear relation fails. The ETV model shows a quartic functional relation and can only generate the observed tortuosity if interstitial viscosity is present. The CCV model is used to analyse the recently described changes in porosity between asleep and awake brain states.

## 1. Introduction

The interstitial space (ISS) of the brain comprises the gaps between the cells. Despite being mostly only tens of nanometers in width, the ISS is a complex domain that has many roles including maintaining an ionic milieu that permits electrical signalling between nerve cells and distributing neuroactive substances, as well as drugs. The ISS is a component of the larger extracellular space; this larger space includes the ventricles and vascular spaces; often the term ‘extracellular space’ is loosely used as a synonym for the ISS. Although the ISS is critical for neurological function it has not received much attention but recently interest has increased [1–4]. Some of this attention followed the identification of a glymphatic system [5,6], which resurfaced a longstanding debate about transport modes in the ISS. Transport of molecules by diffusion is established but movement by advection, i.e. bulk flow, remains controversial. From a transport perspective, brain tissue resembles an unconsolidated porous medium with highly connected pore spaces.

The study of molecular diffusion has been the primary approach to quantifying the ISS. This began with the use of radiotracers in the 1960s and was subsequently refined using the real-time iontophoresis (RTI) and the integrative optical imaging (IOI) methods; most recently super-resolution imaging and single particle tracking are emerging tools [7,8]. The most significant results for this study have been obtained with the RTI method [9,10] using the small tetramethylammonium (TMA) molecule (hydrodynamic diameter < 1 nm). Diffusion analysis confirmed that the ISS occupies about 20% of adult neocortex in anaesthetized rats and mice and in brain slices, which agrees with earlier radiotracer results in larger animals [7]. Apparently, the ISS is reduced to about 14% in the awake mouse cortex [11]; see §4.3. More formally, in a representative elementary volume (REV) of brain tissue, the porosity, *ϕ*, is defined by

1.1 $$\phi =\frac{\mathrm{volume}\;\mathrm{of}\;\mathrm{ISS}\;\mathrm{in}\;\mathrm{REV}}{\mathrm{volume}\;\mathrm{of}\;\mathrm{REV}}.$$

This parameter, also referred to as the extracellular volume fraction, is often denoted by *α* but *ϕ* is used here to follow practice in the porous media literature and avoid confusion with the model parameter ‘*a*’. Thus *ϕ* ∼ 0.2 (i.e. 20%) in the sleeping rodent neocortex. Porosity is larger in the neonatal rat cortex [12] and smaller in some layers of the rat hippocampus [13].

**Figure 1.**
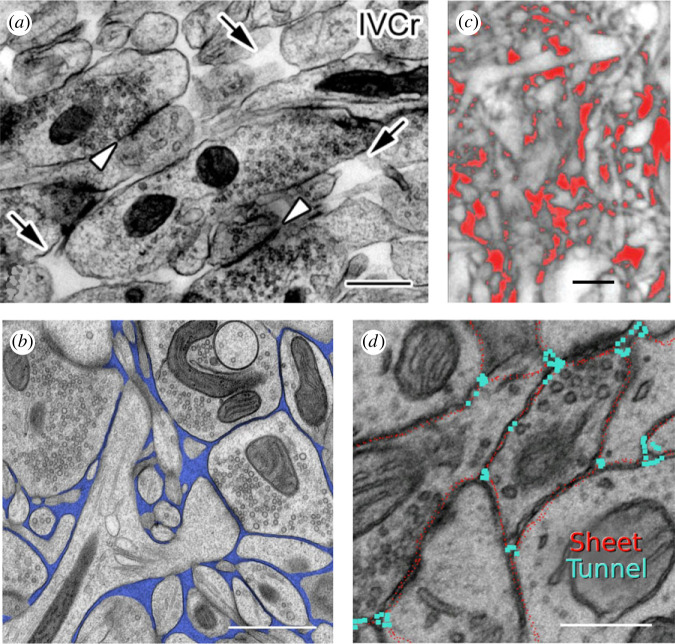
Brain cells and interstitial space (ISS). (*a*) Electron micrograph (EM) of mouse cerebellar molecular layer, prepared with an *in vivo* cryotechnique (IVCr). Arrows show several ISS voids. Open arrowheads indicate synaptic clefts. Scale bar 500 nm. Adapted from [15]. (*b*) EM of cryo-fixed brain tissue from adult mouse cerebral cortex. The ISS is pseudo-coloured blue. Scale bar 1 µm. Adapted from [16]. (*c*) Super-resolution shadow imaging (SUSHI) light microscopy shows part of a mouse hippocampus from an organotypic slice with ISS in red. Scale bar 2 µm. Adapted from [17]. (*d*) Cross-section through the raw reconstruction of three-dimensional block with volume 180 µm^3^ derived from EM images after classical fixation (i.e. ISS is shrunken compared with cryo-fixed tissue). This shows tunnel-like vertices (blue) and sheet-like ISS (red). Scale bar 500 nm. Adapted from [18]. Panels (*a*) and (*d*) copyright: permissions from John Wiley and Sons. Panel (*b*) permission: https://creativecommons.org/licenses/by/4.0/. Panel (c) copyright: permission from Elsevier.

Porosity is a non-dimensional average over all the gaps between the cells contained in the REV. The width of the spaces between cell membranes is less established. Use of quantum dots and fluorescent macromolecules of different sizes combined with restricted diffusion theory suggests that the ‘typical’ width of the ISS may be about 38 nm if the ISS is composed of sheets with parallel walls or 64 nm if the ISS is composed of cylindrical pores [14]. Much of the ISS is composed of narrow sheets between cells, so a typical gap would be approximately 40 nm. However, studies with electron microscopy after cryofixation [15,16] (figure 1*a,b*) and super-resolution optical microscopy [17] (figure 1*c*) indicate that the ISS is not uniform in width but has local expansions or voids. This has been further substantiated by single particle tracking of carbon nanotubes [8].

Electron microscopy using conventionally fixed brain tissue almost eliminates the ISS [15,16], nevertheless Kinney *et al*. [18] were able to use such tissue to reconstruct 180 µm^3^ of rat CA1 hippocampal neuropil and analyse the ISS. They achieved this by algorithmically ‘re-inflating’ the ISS until the porosity was 0.2. This technique cannot restore local expansions of the ISS, but the researchers saw a different type of non-uniformity namely an: ‘…interconnected network of 40–80 nm diameter tunnels [cylindrical pores], formed at the junction of three or more cellular processes, spanned by sheets between pairs of cell surfaces with 10–40 nm width’ [18, p. 448] (figure 1*d*).

In addition to porosity, the geometry of the ISS affects another parameter, geometrical tortuosity, *τ*_g_, and the value of this parameter refines the choices for models of ISS structure. Diffusion analysis enables *τ*_g_ to be defined and measured. This parameter is usually denoted by *λ* in the neuroscience literature but *τ* is employed here to again conform to porous media usage. Geometrical tortuosity is a measure of the hindrance imposed by the structure of the ISS on a diffusing molecule and is defined here as [9]

1.2 $$\tau_{g}=\sqrt{D/D^{*}},$$

where *D* is the free diffusivity of a molecule (i.e. that is measured in water or dilute agarose gel) and D* is the effective diffusivity measured in brain tissue with RTI or other methods. Note that there are other definitions of tortuosity in the porous media literature. The most common value of *τ*_g_ obtained in the rodent neocortex, and several other brain regions, using a small molecule like TMA, is *τ*_g_ ∼ 1.6 meaning that D* is about 40% smaller than *D* [7].

Tortuosity is not a simple parameter. This paper will regard tortuosity as a function of the average path-length taken by an infinitesimally small molecule moving in the pore space of a porous medium between any two points compared with the path length defined by the straight-line distance between those two points. The porous medium is defined in strictly geometrical terms, so other factors that might affect molecular movement, such as surface absorption or chemical reactions, are absent. The difficulty with this definition is measuring the average path-length in the medium. Hydrodynamics, geophysics, materials science, electrochemistry and other disciplines have wrestled with this problem, [19,20]. Here it will be assumed without proof that equation (1.2) provides a consistent measure of tortuosity.

The interpretation of diffusion measurements would be affected if the medium filling the ISS had a substantial viscosity. The interstitial fluid resembles cerebrospinal fluid, which has a viscosity close to that of water [21], so this can be neglected, but there might be a viscous contribution from the extracellular matrix that is present in the ISS. One study attempted to measure ISS viscosity [22] using time-resolved fluorescence anisotropy imaging in the rat hippocampus employing a fluorescent probe molecule of similar size to TMA. The study reported a 30% decrease in the rotational diffusivity of the probe, which equates to a viscous tortuosity component of *τ*_viscous_ = 1.2. If this value is accepted and is applicable to other brain regions, then this non-geometrical component would need to be factored out. Tortuosities are multiplicative [23], so if the measured tortuosity is typically *τ*_measured_ = 1.6 then *τ*_measured_ = *τ*_g_ × *τ*_viscous_ and *τ*_g_ would actually be 1.333. Both *τ*_g_ = 1.6 and *τ*_g_ = 1.333 will be modelled here, although the viscosity value is derived from limited measurements.

By contrast to the effect of viscosity, geometrical tortuosity is legitimately increased through the contribution of non-uniformity of the ISS [24–26] that creates dead-space microdomains. For example, if a diffusing molecule enters a local ISS expansion, then it is more likely to collide with the wall of the expansion than to enter a narrower channel connecting to the rest of structure, and so the molecule does not advance as quickly as when the entire medium is connected by uniform channels. Other examples of dead spaces have been described [25,26]. This study will model both an ISS with local voids at the corners of cubic cells and one with a network of tunnels located at the cube edges, both connected by a syncytium of narrower sheets representing the predominant space between cell membranes. The tortuosities of these models will be measured by Monte Carlo simulation of diffusion in the ISS. Other models are possible, including the ‘missing cubic cells’ model [27]; that model will be further analysed in future work.

This paper will show that the width of the basic cubic cell typically varies between about 0.5 and 2.6 µm, which is smaller than a typical neuronal cell body. Rather, it should be understood that such dimensions represent the cross-sections of dendrites, unmyelinated axons, and glial processes forming the neuropil of the neocortex and elsewhere, as seen in electron micrographs and in super-resolution optical images (figure 1). An alternative perspective is that the cubic cells provide a means of distributing the voids, which will have the same spacing as the width of the basic cubic cells plus width of the sheet component of the ISS.

## 2. Models based on cubic cells

The models were constructed by creating an ensemble of identical cells and packing them together with a defined spacing to obtain the required porosity. The simplest arrangement used cubical cells and it is clear that these can fill three-dimensional space. A more complex cell shape that packs three-dimensional space is the truncated octahedron. Going beyond these two mono-cell models requires combinations of cells of different shapes to pack three-dimensional space. In an extensive study, Tao & Nicholson [28] showed that all these models generate similar tortuosities and Hrabe *et al*. [29] showed that certain random polyhedra give the same result. The key properties of all these ensembles were that the cells were convex in shape and the distance between adjacent cell surfaces was the same. Because all these models produce the same results as an ensemble of cubes, only cubes need be considered. The main parameters used in this paper are listed in table 1.

**Table 1.** Main parameters.

parameter	unit	description	typical experimental value [reference]
*ϕ*		porosity (ISS volume fraction)	0.18–0.25 [7]
*τ*_g_		geometrical tortuosity, $\tau_{g}=\sqrt{D/D^{*}}$	1.5–1.7 [7]
*D*	cm^2^ s^−1^	free diffusivity of tetramethylammonium (TMA)	1.11 × 10^−5^ at 37°C [7]
D*	cm^2^ s^−1^	effective diffusivity in brain measured with TMA	3.84 × 10^−6^–4.93 × 10^−6^ at 37°C [7]
*τ*_viscous_		component of experimental tortuosity possibly attributable to viscosity	1.2 [22]
*w*	nm	sheet half-width	5–32 [14,18]
*a*	μm	basic cubic cell half-width	
*b*	μm	void width	
*p*	μm	bounding cube half-width, *p* = *a* *+* *w*	
*V*_a_	μm^3^	volume of basic cubic cell, *V*_a_ = 8*a*^3^	
*V*_p_	μm^3^	volume of bounding cube, *V*_p_ = 8*p*^3^	
*V*_s_	μm^3^	volume of sheets surrounding basic cubic cell, *V*_s_ = *V*_p_ − *V*_a_	
*V*_v_	μm^3^	volume of all voids associated with basic cubic cell	
*Ω*		*Ω* = *V*_v_/*V*_s_	
*A*		*a* = *Aw*, see equation (2.12).	
*B*		*b* = *Ba*, see equation (2.15), only true for CCV	
*τ*_g00_		tortuosity for ensemble of cubes when voids are absent (*Ω* = 0), see equation (2.2)	
*τ*_g0_		constant term of fitting polynomials	
*m*_1_ – *m*_4_		coefficients of fitting polynomials	

### 2.1. Cubic cells pack three-dimensional space but do not generate the observed tortuosity

In an ensemble of cubic cells with a regular packing the ISS takes the form of a set of intersecting sheets. This model has been extensively studied with Monte Carlo simulations [28,30], so only a brief summary will be given. The simulations reveal a remarkably simple relationship between *ϕ* and *τ*_g_,

2.1 $$\tau_{g}=\sqrt{\frac{3-\phi }{2}}.$$

This expression is related to the famous result for the effective resistance of an ensemble of loosely packed spheres (the dilute ensemble) published by Maxwell in 1891 [31]. The packed cubic cell problem was revisited by Nicholson and Kamali-Zare [30] using high-precision Monte Carlo simulations and a small correction suggested,

2.2 $$\tau_{g}=\sqrt{\frac{3-\phi }{2}}+0.07\phi (1-\phi ).$$

Equations (2.1) and (2.2) lack physical dimensions so the cells can be any size unless the width of the ISS is fixed. For a fixed width 2*w* and a cubic cell of side 2*a*

2.3 $$\frac{a}{w}=\frac{\gamma }{1-\gamma },$$

where $\gamma =\sqrt[{3}]{\left(1-\phi \right)}$. If 2*w* = 40 nm (see Introduction) and *ϕ* = 0.2 then *a* = 0.259 µm.

Equations (2.1) and (2.2) are valid for 0 ≤ ø ≤ 1 so it follows that the maximum value of *τ*_g_ occurs as *ϕ* → 0 when $\tau_{g}=\sqrt{3/2}=1.225$. This value is substantially smaller than the tortuosity observed experimentally (*τ*_g_ ∼ 1.6) and less than the tortuosity even considering possible viscosity (*τ*_g_ ∼ 1.333), so the brain is not a collection of equally spaced cubes! Thus a more complex ISS geometry is required that will of necessity violate the conditions that cell surfaces are uniformly spaced apart and/or the cells are convex. Such violation will create dead space potentially leading to an increase in geometrical tortuosity.

### 2.2. General relations for cells, sheets and voids

**Figure 2.**
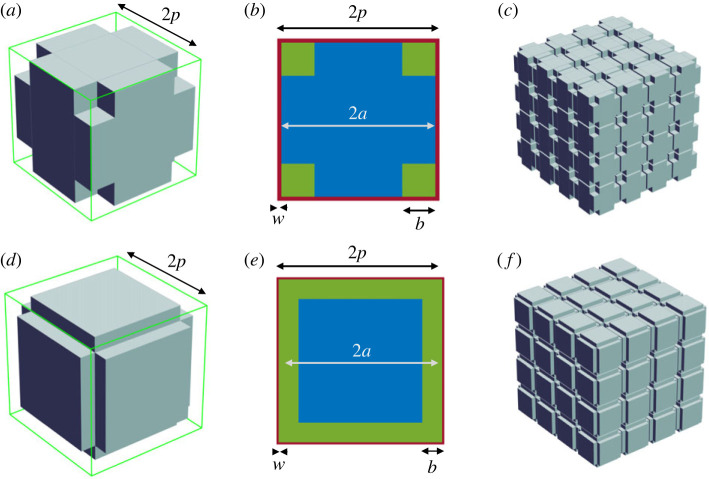
Model cells. (*a*) Corner cubic void (CCV) model. Frame of side 2*p* indicated in green defines the bounding cube. (*b*) View of two-dimensional surface looking from top. Basic cell (blue) has side 2*a* and voids (green) have sides *b*. ISS sheet of width *w* shown in red. Note that ISS lies outside both the basic cell and the voids but within the bounding cube. (*c*) Ensemble of CCV cells. (*d*) Edge tunnel void (ETV) model. Frame of side 2*p* indicated in green. (*e*) View of two-dimensional surface of ETV cell looking from top. Basic cell (blue) has side 2*a*, and voids (green) have tunnel of width *b* and length 2(*a* – *b*) at each edge plus a cubic void of width *b* at each corner. Sheet of ISS of width *w* is shown in red. (*f*) Ensemble of ETV cells. For both CCV and ETV models the cells were generated with *ϕ* = 0.2, *w* = 20 nm; *a* and *b* were chosen so that the simulation gave *τ*_g_ = 1.333. In panels (*c*) and (*f*) ensemble of 4 × 4 × 4 cells shown, but 32 × 32 × 32 or 64 × 64 × 64 cells used in actual simulations.

The primary model considered here is a basic cubic cell, of width 2*a*, and volume *V*_a_, either modified by the formation of cubic voids at each corner, the corner cubic void (CCV) model (figure 2*a*,*b*), or by the formation of tunnels along each edge of the basic cube, the edge tunnel void (ETV) model (figure 2*d*,*e*). The sum of all voids associated with a basic cubic cell is *V*_v_. Each modified cell is surrounded by sheets of ISS of width *w* so that two adjacent cells are separated by a distance 2*w*, except where the voids are present, and the total volume of sheets around a cubic cell is *V*_s_ (figure 2*b*,*e*). The outer boundary of the enclosing sheets around each cell forms a bounding frame of width 2*p* = 2(*a* + *w*) as shown in figure 2*a*,*d*. This bounding frame defines a structure in the shape of a cube of width 2*p* and volume *V*_p_, and these bounding cubes form the ensemble that packs three-dimensional space (figure 2*c*,*f*).

It is useful to define a new dimensionless variable

2.4 $$\Omega =\frac{V_{v}}{V_{s}}.$$

Thus *Ω* is the ratio of total void volume *V*_v_ to the sheet volume *V*_s_ in the bounding cube. Note that the exterior of each void is covered by a sheet of ISS that is not included in the void volume *V*_v_ (figure 2*b*,*e*) but is included in the total ISS porosity. This delineation enables an appropriate model as *V*_v_ → 0 that will just leave the ISS sheets surrounding a basic cube.

From the definition of *ϕ* given in equation (1.1)

2.5 $$\phi =\frac{V_{s}+V_{v}}{V_{p}}.$$

Usually we specify *ϕ*, *V*_a_ (through the value of *a*) and *V*_p_ (through the values of *a* and *w*), so, bearing in mind that *V*_s_ = *V*_p_ − *V*_a_, an expression for *Ω* may be obtained from equations (2.4) and (2.5)

2.6 $$\Omega =\frac{\phi V_{p}-V_{s}}{V_{s}}=\frac{V_{a}-(1-\phi )V_{p}}{V_{p}-V_{a}}.$$

Now, *V*_a_ = (2*a*)^3^ and *V*_p_ = (2*p*)^3^, so from equation (2.6)

2.7 $$\Omega =\frac{a^{3}-(1-\phi )p^{3}}{\;p^{3}-a^{3}},$$

furthermore, because *p* = *a* + *w*, this may be written as

2.8 $$\Omega =\frac{{\left(a/w\right)}^{3}-(1-\phi ){\left((a/w)+1\right)}^{3}}{{\left((a/w)+1\right)}^{3}-{\left(a/w\right)}^{3}}.$$

An approximation to equation (2.8) may be useful. Expanding the cubic terms and recognizing that *a/w* ≫ 1

2.9 $$\Omega \approx \frac{{\left(a/w\right)}^{3}-(1-\phi )({\left(a/w\right)}^{3}+3{\left(a/w\right)}^{2})}{3{\left(a/w\right)}^{2}}.$$

This simplifies to

2.10 $$\Omega \approx \frac{a}{w}\frac{\phi }{3}-(1-\phi ),$$

so, if *ϕ* is fixed then *Ω* should be approximately proportional to *a/w*.

Another useful relation derived from equation (2.8) is

2.11 $$\frac{w}{a}={\left[\frac{1+\Omega }{1+\Omega -\phi }\right]}^{1/3}-1.$$

Equations (2.8)–(2.11) imply that any combination of *a/w* that has the same value will result in the same *Ω*. Equation (2.11) leads immediately to an expression enabling *a* to be calculated from *w*

2.12 $$a=Aw,\;\text{ where }\;A={\left\{{\left[\frac{1+\Omega }{1+\Omega -\phi }\right]}^{1/3}-1\right\}}^{-1}.$$

Note that all these relations are independent of specific void geometry; volumetric aspects of void geometry are captured in *ϕ* and *Ω*. Equations for explicit void geometry follow in the next sections.

### 2.3. Corner cubic void model

To construct models the void geometry must be specified. Starting with equation (2.5),

2.13 $$V_{v}=V_{a}-(1-\phi )V_{p},$$

and substituting *V*_a_ = 8*a*^3^, *V*_p_ = 8(*a* *+* *w*)^3^ and for a CCV model with void width *b*, *V*_v_ = 8*b*^3^, then

2.14 $$b=a{\left\{1-(1-\phi ){\left[1+\frac{w}{a}\right]}^{3}\right\}}^{1/3}.$$

Combining with equation (2.11) this may also be expressed as

2.15 $$b=Ba=ABw,\;\text{ where }\;B={\left\{\frac{\Omega \phi }{1+\Omega -\phi }\right\}}^{1/3}.$$

It is also useful to note that

2.16 $$\phi =1-\frac{\left(a^{3}-b^{3}\right)}{\;p^{3}}.$$

### 2.4. Edge tunnel void model

Again equation (2.13) is the starting point; *V*_a_ and *V*_p_ retain their definitions but *V*_v_ has a more complex definition (figure 2*d,e*). Each cube has 12 edges, each with a tunnel with volume 2(*a* − *b*)*b*^2^. In addition there are eight corners, each with a cubic void of volume *b*^3^. Putting all this together, for the ETV model, *V*_v_ = 8(3*a* + 2*b*)*b*^2^. Inserting this in equation (2.13) and re-arranging

2.17 $$2b^{3}-3ab^{2}+\{a^{3}-(1-\phi ){\left(a+w\right)}^{3}\}=0.$$

The variables *a*, *w* and *ϕ* are specified so the term in curly brackets is a constant. This cubic equation is solved for *b* yielding three roots and the physically plausible one selected. Again, note that

2.18 $$\phi =1-\frac{\left(2b^{3}-3ab^{2}+a^{3}\right)}{\;p^{3}}.$$

## 3. Methods

The Monte Carlo simulations employed the program MCell, version 3.4, ([32]; www.mcell.org) (a few simulations used earlier versions of MCell) using Windows running on a quad-core Intel-based workstation with 32 gigabytes of memory. The derivation of the geometry parameters for the MCell script (see electronic supplementary material for script examples) and analysis of the MCell output and construction of three-dimensional figures used custom programs written in MATLAB (MathWorks, Natick, MA, USA). Curves were fitted to polynomials using the MATLAB function polyfit.m and roots were found with roots.m. The coefficient of determination was calculated as $R^{2}=1-{\left(\left\|\mathrm{residuals}\right\|/\left\|\left(Y-\mathrm{mean}(Y)\right)\right\|\right)}^{2}$ where *Y* is the vector of the data and ‖‖ is the norm; the relevant parameters were output by polyfit.m.

Values of *ϕ* and *w* were specified; in addition one more parameter was needed. If the parameter was *a*, then *b* was calculated from equation (2.14) or equation (2.17). If *Ω* was specified, then *a* was calculated from equation (2.12) and from this value *b* calculated as before. Knowing *a*, *b* and *w* an ensemble of CCV or ETV cells could be programmed using the MCell scripting language (see electronic supplementary material).

Most simulations employed 32 × 32 × 32 cells but some used 64 × 64 × 64 cells to ensure that molecules remained within the ensemble when the simulations needed to run for extended times to reach a stable tortuosity value. A total of 25 000 molecules were released instantaneously from a point-source in the centre of the void in the middle of the ensemble. The time step, Δ*t*, was chosen so that mean radial displacement ${\bar{l}}_{r}=4\sqrt{D\Delta t/\pi }$ (equation (4.6) in [32]) was much smaller than 2*w*. In most simulations Δ*t* = 20 ns. Test simulations were made with smaller Δ*t* to check that this did not alter the results.

Within the ISS, the molecules moved with a diffusivity *D*. Two values were used. For models where interstitial viscosity was assumed absent, the free diffusion value for tetramethylammonium at 37°C, *D* = 1.11 × 10^−5^ cm^2^ s^−1^ [7], was used. Simulations incorporating interstitial viscosity employed the value for *D* appropriate for *τ*_viscous_ = 1.2; this value was *D* = 7.71 × 10^−6^ cm^2^ s^−1^. After the molecular distribution in the simulation reached a steady state (see §§4.1.1 and 4.2.1) a value of D* was obtained. Tortuosity, *τ*_g_, was calculated from this D* and the value of *D* employed in the simulation, using equation (1.2). Note that the steady state value of *τ*_g_ is independent of value of *D* used in the simulation. The value of D* was found by computing the mean square distance from the point source of every diffusing molecule at a chosen time *t* [30] using the equation

3.1 $$D^{*}=\frac{\left\langle r^{2}\right\rangle }{6t},$$

where 〈*r*^2^〉 is mean square of the radial distance of each molecule from the release point (*r* = 0). Prior to reaching a steady state, D* was also computed according to equation (3.1) but, because the value was time-dependent, it did not represent a ‘classic’ diffusivity at these early times and the computed *τ*_g_ was also time-dependent. After *τ*_g_ reached a steady state, it was averaged over an appropriate set of output iterations. The typical duration of the simulation was 0.01–0.05 s. A check was made to see that no molecules had left the ensemble before the end of the simulation.

One Monte Carlo simulation with one random number seed (usually seed #1) was run for each data point (see later plots). In §4.3, where only two data points were required, five simulations with seeds #1–#5 were run and the resulting tortuosities averaged but the variation between runs was small. Therefore one seed was deemed adequate for most of the work, especially as more than 100 simulations were run, each averaging about 5 h of computer time. This type of problem lends itself to periodic boundary conditions, but this approach was not employed because of the added complexity and the availability of a workstation with adequate memory enabled the present simpler three-dimensional implementation (see [30]).

## 4. Results

### 4.1. Tortuosities for cubes with corner cubic voids

The simulations for this study began in 2017 following earlier preliminary studies with a CCV model [33]. The construction of the three-dimensional model within MCell has been changed from that in the preliminary studies to enable more flexible geometries, including the ETV model, and is illustrated in the MCell scripts in the electronic supplementary material.

The strategy for the simulations was to select combinations of three realistic values of *ϕ* (0.1, 0.2 and 0.4) and three values of *w* (10, 20 and 40 nm) giving nine parameter combinations. MCell simulations for each combination were made with a sequence of basic cube sizes (i.e. a sequence of *a*-values) to obtain *τ*_g_ values. A linear relation was observed between $\tau_{g}^{2}$ and *a* for any given pair of *ϕ*, *w* values, so there were nine linear relationships. Because $\tau_{g}^{2}$ is dimensionless but *a* is not, this was an unsatisfactory relationship. Further analysis suggested a linear relationship between $\tau_{g}^{2}$ and the dimensionless parameter *a/w*; this resulted in just three linear relationships, one for each value of *ϕ*. However, because *a/w* was geometry specific a more general dimensionless variable was sought. It was discovered that the ratio of void volume to sheet volume (*Ω*) was linearly related to $\tau_{g}^{2}$. Later analysis (equation (2.10)) showed that, to a good approximation, *a/w* and *Ω* are linearly related for the CCV geometry.

#### 4.1.1. Evolution of tortuosity with time for corner cubic void model

**Figure 3.**
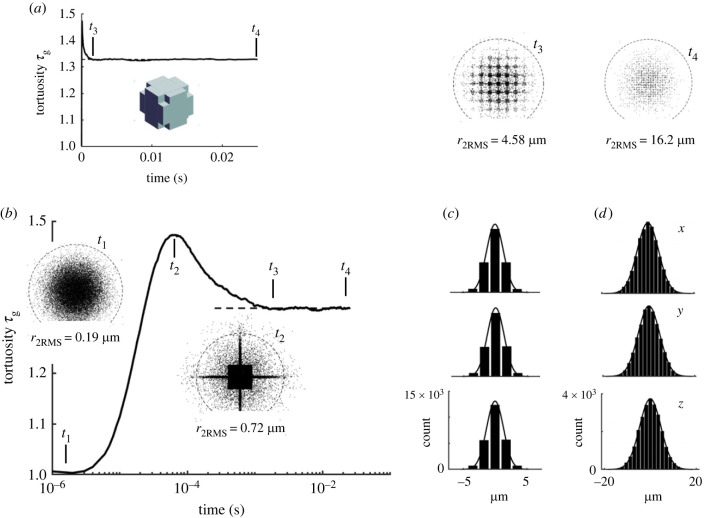
Time evolution of tortuosity in 64 × 64 × 64 ensemble of CCV cells (*a*) Linear timescale. There is an initial peak in tortuosity after which it settles to a steady state by time *t*_3_ that continues to end of simulation at *t*_4_. Inset shows model of the CCV cell. (*b*) Logarithmic timescale. At *t*_1_ = 2 µs molecules are mostly undergoing free diffusion after release from centre of ensemble, as seen in snapshot. Dashed circle in snapshot drawn at 2 × RMS distance calculated for population of molecules at *t*_1_. Snapshot at *t*_2_ = 60 µs shows molecules have filled central cavity and adjacent sheets of ISS and have reached the next set of cavities and tortuosity reaches a peak. At *t*_3_ = 2 ms, tortuosity attains a steady state and snapshot shows extent of molecular penetration into ensemble. Steady state persists until simulation ends at *t*_4_ = 25 ms. (*c*) Histograms of *x*, *y* and *z* coordinates of molecules at *t*_3_ with an independently generated Gaussian curve. (*d*) Histograms at *t*_4_, the end of the simulation. For this simulation *ϕ* = 0.2, *a* = 0.416 µm, *b* = 0.179 µm, *w* = 20 nm (so *p* = 0.436 µm); histogram bin width is 4*p* = 1.744 µm. ‘count’ is number of molecule coordinates.

The geometry depicted in figure 2*a–c* is used to show how the diffusion process evolves. From the extensive simulations described later in §4.1.2, it became apparent that a CCV model with *a* = 0.416 µm, *b* = 0.1785 µm and *w* = 20 nm (figures 2*a*,*b*; 3*a* inset) results in an ensemble with *ϕ* = 0.2 and an MCell simulation that produces *τ*_g_ = 1.328, close to the expected value for a viscous ISS.

Figure 3*a* shows how the calculated tortuosity evolves with linear time. The apparent tortuosity rapidly rises to a peak, which is barely discernable in figure 3*a*, and then falls more slowly to a steady state value by time *t*_3_ = 0.002 s (2 ms) that persists until the end of the simulation at *t*_4_ = 0.025 s. The temporal evolution is more evident in figure 3*b* where time is plotted on a logarithmic scale.

The distributions of molecules at four times *t*_1_–*t*_4_, are visualized by taking a slice through the entire ensemble centred on the *x–y* plane with a thickness in the z-axis of *z* *=* ± *p*. The molecules in this slice are then projected on the *x–y* plane. This provides a snapshot of all the molecules in the central void and those that have spread out in the *x-y* sheet (thickness 2*w*) centred on *z* = 0 as well as those molecules moving in the orthogonal *x–z* and *y–z* sheets contained in the vertical extent of the slice (i.e. ± *p*).

The first phase of molecular spreading occurs immediately after the molecules are released and move in the cavity at the centre of the ensemble that is formed from four cubic voids each of side *b* = 0.1785 µm so the molecules have to move at least this far to encounter a cell wall. A snapshot of the projected molecules in the slice described above is shown in the inset for time *t*_1_ = 2 µs. The inset shows the uniform distribution of molecules; to provide a distance scale, the dashed circle around the molecules has a radius of twice the root-mean-square distance value of all the molecules at this time, which is *r*_2RMS_ = 0.19 µm. Most molecules are less than *r*_2RMS_ from the release site and are still undergoing free diffusion in the void and consequently *τ*_g_ = 1.

As the molecules reach the cell walls bounding the central void, many are reflected but some find the entrances to the system of sheets emanating from the cavity. This reflection causes a rapid rise in tortuosity because the molecules are now more hindered. The tortuosity peaks at *t*_2_ = 60 µs and a snapshot at this time shows the central cavity as a dense black aggregate of molecules and the sheets leaving the cavity are outlined by the molecules moving within them. Some molecules are in the sheet associated with the *x–y* plane and appear as a lighter sprinkling of molecules. The dashed circle is now at *r*_2RMS_ = 0.72 µm showing the magnification of this snapshot is reduced compared with that at *t*_1_. The distance from the middle of the central cavity to the middle of the next cavity is 2*p* = 0.872 µm; thus this snapshot is taken at a time when some molecules have traversed the initial set of sheets and are encountering the next set of voids. This accounts for the peak in tortuosity, because from now on those molecules entering the new set of voids will move more easily until they encounter the next set of walls. Note that values of *τ*_g_ calculated during this rising phase are not ‘real’ tortuosities because the computed D* on which they are based changes with time.

The molecules continue to move through the cellular ensemble and their trajectories increasingly average over sheets and voids, so the tortuosity declines after time *t*_2_. Tortuosity does not fall indefinitely, however, instead after *t*_3_ = 2 ms, it reaches a steady state and remains constant until the end of the simulation at *t*_4_ = 25 ms. The snapshot at *t*_3_ reveals how much of the structure must be explored by the molecules before an effective diffusivity, D*, is established. The validity of D* as a classic diffusivity is confirmed by the histograms and independent Gaussian curve fits in figure 3*c* at time *t*_3_. The snapshot at *t*_3_ suggests that molecules must explore a distance of about three cells out from the release site; this is 6*p* = 2.62 µm, (a block of about 200 cells) to achieve a stable distribution. The dashed circle in this snapshot is at *r*_2RMS_ = 4.58 µm. Finally, the snapshot at *t*_4_ = 25 ms shows the molecular distribution in the slice at the end of the simulation with *r*_2RMS_ = 16.2 µm and the histograms are shown in figure 3*d*. The cellular structure of the ensemble is barely discernable in the snapshot.

#### 4.1.2. Summary of analysis of tortuosities of corner cubic void models with *ϕ* = 0.1, 0.2 and 0.4

**Figure 4.**
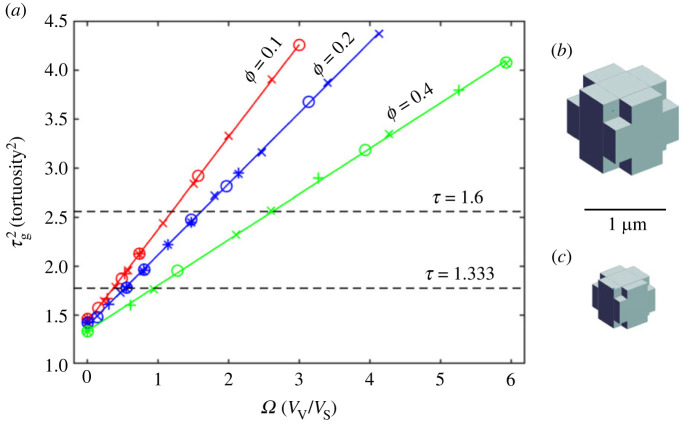
Results for CCV model with porosity in the normal range. (*a*) Porosities *ϕ* = 0.1, 0.2 and 0.4 were combined with ISS sheet half-widths *w* = 10, 20 and 40 nm. For each combination of *ϕ* and *w*, a sequence of *a*-values was chosen, and *b* calculated from equation (2.14), the cellular geometry constructed, and MCell simulations run to obtain a steady state value of $\tau_{g}^{2}$. Values of $\tau_{g}^{2}$ were plotted against *Ω*, calculated from equation (2.8). See text for further discussion. Symbols indicate relation of data point to *w*-value; ‘o’ designates simulations with *w* = 10 nm, ‘×’ with *w* = 20 nm, ‘ + ’ with *w* = 40 nm. (*b*) CCV model that gives *τ*_g_ = 1.603 ($\tau_{g}^{2}=2.569$) for *ϕ* = 0.2, *w* = 20 nm. (*c*) CCV model that gives *τ*_g_ = 1.328 ($\tau_{g}^{2}=1.765$= ) for *ϕ* = 0.2, *w* = 20 nm. See table 4 for cell dimensions. Models in panels (*b*) and (*c*) have correct relative scales.

The parameters for the model described in the previous section were taken from a sequence of 50 MCell simulations of CCV cells; the sequence is summarized in figure 4*a*. The most impressive feature is that, for a given value of *ϕ* ≤ 0.4, in the range of *τ*_g_ and *Ω* specified, $\tau_{g}^{2}$ is a linear function of *Ω*,

4.1 $$\tau_{g}^{2}=\tau_{g0}^{2}+m_{1}\Omega ,$$

where $\tau_{g0}^{2}$ is the value of $\tau_{g}^{2}$ when the voids are absent (*Ω* = 0) and only sheets are present. The fitting parameters are shown in table 2.

**Table 2.** Fitting parameters for data shown in figure 4. $\tau_{g00}^{2}$ was independently calculated from equation (2.2). *R*^2^ coefficient of determination (see Methods). *n* number of simulations for each value of *ϕ*.

*ϕ*	$\tau_{g00}^{2}$	$\tau_{g0}^{2}$	*m*_1_	*R*^2^	*n*
0.1	1.465	1.437	0.941	0.9996	19
0.2	1.427	1.399	0.722	0.9996	26
0.4	1.339	1.345	0.463	0.9996	14

**Table 4.** Solutions to equation (4.1) for *w* = 20 nm.

	*τ*_g_ = 1.6, *w* = 20 nm			*τ*_g_ = 1.333, *w* = 20 nm		
	*Ω*	*a* (µm)	*b* (µm)	*Ω*	*a* (µm)	*b* (µm)
*ϕ* = 0.1	1.193	1.276	0.491	0.361	0.776	0.238
*ϕ* = 0.2	1.608	0.742	0.379	0.523	0.416	0.179
*ϕ* = 0.4	2.624	0.503	0.346	0.933	0.249	0.155

Equation (4.1) can be immediately solved to yield the *Ω* values for *τ*_g_ = 1.6 and *τ*_g_ = 1.333 for a chosen value of *ϕ* (intercepts with dashed lines in figure 4*a*) and the corresponding values of *a* and *b* obtained using equations (2.12) and (2.15) for a given value of *w*. These results are shown in tables 3–5. The large variation in size of the basic cell (half width *a*) is evident. Figure 4*b* shows the basic cell corresponding to *τ*_g_ = 1.6 while figure 4*c* shows the cell associated with *τ*_g_ = 1.333, both with *w* = 20 and *ϕ* = 0.2. The cells have the correct relative sizes, showing that it requires a smaller cell to generate the smaller geometric tortuosity.

**Table 3.** Solutions to equation (4.1) for *w* = 10 nm.

	*τ*_g_ = 1.6, *w* = 10 nm			*τ*_g_ = 1.333, *w* = 10 nm		
	*Ω*	*a* (µm)	*b* (µm)	*Ω*	*a* (µm)	*b* (µm)
*ϕ* = 0.1	1.193	0.638	0.246	0.361	0.388	0.119
*ϕ* = 0.2	1.608	0.371	0.190	0.523	0.208	0.089
*ϕ* = 0.4	2.624	0.252	0.174	0.933	0.124	0.070

**Table 5.** Solutions to equation (4.1) for *w* = 40 nm.

	*τ*_g_ = 1.6, *w* = 40 nm			*τ*_g_ = 1.333, *w* = 40 nm		
	*Ω*	*a* (µm)	*b* (µm)	*Ω*	*a* (µm)	*b* (µm)
*ϕ* = 0.1	1.193	2.552	0.982	0.361	1.553	0.475
*ϕ* = 0.2	1.608	1.484	0.759	0.523	0.833	0.357
*ϕ* = 0.4	2.624	1.006	0.692	0.933	0.498	0.311

#### 4.1.3. Summary of analysis of tortuosities of corner cubic void models with *ϕ* = 0.6, 0.8 and 0.9

**Figure 5.**
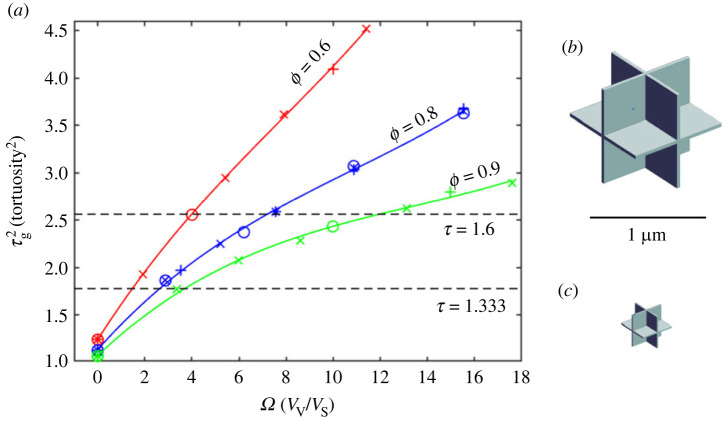
Results for CCV models with porosity above the normal range. (*a*) Porosities *ϕ* = 0.6, 0.8 and 0.9 were combined with ISS sheet half widths *w* = 10, 20 and 40 nm. Procedures follow those described for figure 4. Symbols indicate relation of data point to the chosen *w*-value; ‘o’ designates simulations with *w* = 10 nm, ‘×’ with *w* = 20 nm, ‘ + ’ with *w* = 40 nm. (*b*) Geometry of CCV model corresponding to the intercept of the *ϕ* = 0.9 line (green) with $\tau_{g}^{2}=2.56$ (*τ*_g_ = 1.6) (*c*) Geometry of CCV model corresponding to the intercept of the *ϕ* = 0.9 line (green) with $\tau_{g}^{2}=1.777$ (*τ*_g_ = 1.333). Cell dimensions described in the text. Models in panels (*b*) and (*c*) have correct relative scales.

These porosities are much higher than normally found in brain tissue and so the findings will only be presented briefly. Results of 35 MCell simulations are shown in figure 5*a*. The data points for a specified *ϕ* no longer can be fitted with a straight line but instead require a third-order polynomial,

4.2 $$\tau_{g}^{2}=\tau_{g0}^{2}+m_{1}\Omega +m_{2}\Omega^{2}+m_{3}\Omega^{3}.$$

The fitted parameters are shown in table 6.

**Table 6.** Fitting parameters for data shown in figure 5. See tables 1 and 2 for description of parameters.

*ϕ*	$\tau_{g00}^{2}$	$\tau_{g0}^{2}$	*m*_1_	*m*_2_	*m*_3_	*R*^2^	*n*
0.6	1.237	1.237	0.391	−0.0180	0.000774	0.9999	9
0.8	1.124	1.132	0.279	−0.0145	0.000447	0.9993	16
0.9	1.063	1.069	0.236	−0.0132	0.000328	0.9983	10

In accord with equations (2.8)–(2.10), any ratio of *a*/w (in the range *w* ≤ 40 nm) that gives the same value of *Ω* lies on the fitted lines.

The pathological porosities represented in figure 5*a* lead to correspondingly abnormal cellular geometry as shown in figure 5*b*,*c*. The parameters for these figures were derived by obtaining the roots of the cubic equation defined in the last row of table 6. The relevant root is indicated by the intercepts of the *ϕ* = 0.9 plot with the $\tau_{g}^{2}$ dashed line. For figure 5*b* the root for $\tau_{g}^{2}=2.56$ (*τ*_g_ = 1.6) is *Ω* = 11.886 resulting in *a* = 0.819 µm, *b* = 0.788 µm. For figure 5*c* the root for $\tau_{g}^{2}=1.777$ (*τ*_g_ = 1.333) is *Ω* = 3.692 resulting in *a* = 0.272 µm, *b* = 0.260 µm.

### 4.2. Tortuosities for cubes with edge tunnel voids

The study of Kinney *et al*. [18] suggested that the ISS geometry was composed of sheets and tunnels (figure 1*d* and Introduction). In this paper the sheet-and-tunnel paradigm is realized as the ETV model and analysed to see if can provide experimentally measured tortuosities. The ETV model has rectangular tunnels along the edges of a basic cubic cell; this has the potential to increase tortuosity through the disparity in the widths of the sheets compared with the tunnels.

#### 4.2.1. Evolution of tortuosity with time for edge tunnel void model

**Figure 6.**
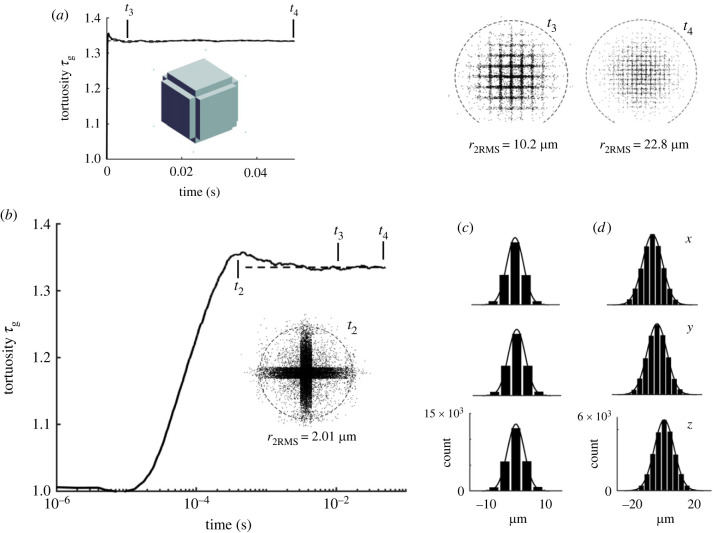
Time evolution of tortuosity for 64 × 64 × 64 ensemble of ETV cells. (*a*) Linear timescale. There is an initial peak in tortuosity then values settle to a steady state by time *t*_3_ and beyond. Inset shows a model of the ETV cell. (*b*) Logarithmic timescale. The early distribution of molecules (0–10 µs) while they are undergoing free diffusion not shown but would resemble the snapshot at *t*_1_ in figure 3. At *t*_2_ = 0.4 ms molecules occupy the central cavity and adjacent sheets of ISS and reach the next set of cavities, as shown in the snapshot for *t*_2_ at peak tortuosity. Dashed circle in snapshot drawn at 2 × RMS distance calculated for population of molecules at *t*_2_ to provide scale. At *t*_3_ = 10 ms tortuosity attains a steady state that persists until the end of the simulation at *t*_4_ = 50 ms. Snapshots at *t*_3_ and *t*_4_ show extent of molecular penetration into ensemble. (*c*) Histograms of *x*, *y* and *z* coordinates of molecules at *t*_3_ with an independently generated Gaussian curve. (*d*) Histograms at *t*_4_. For this simulation *ϕ* = 0.2, *a* = 0.935 µm, *b* = 0.226 µm*, w* = 20 nm (so *p* = 0.955 µm). For histograms, bin width is 4*p* = 3.819 µm. ‘count’ is number of molecule coordinates.

The ETV model is shown in figure 2*d* and in the inset to figure 6*a*. Again, note that the whole cell is enclosed in sheets of ISS (figure 2*e*). Figure 6 illustrates the evolution of the molecular distribution for an ensemble of ETV model cells.

Figure 6*a*, and especially figure 6*b*, show that the time-dependent phase of the tortuosity exhibits a peak at *t*_2_ = 400 µs, the time when molecules are just entering the second set of voids (see snapshot labelled ‘*t*_2_’). This peak behaviour is like that seen for CCV with same porosity (figure 3*a*,*b*). At *t*_3_ = 10 ms the tortuosity reaches a steady state, and the snapshot shows that most of the molecules are confined within an ensemble of about 6 × 6 × 6 cells, and the histograms in figure 6*c* reveal that a classic diffusion condition exists. At *t*_4_ = 50 ms the diffusion condition is completely evident in the histogram (figure 6*d*) but some structure is still visible in the snapshot showing the molecular distribution.

Thus the behaviour of the molecules in the ETV model resembles the CCV model. The time of peak in the ETV model (*t*_2_ = 0.4 ms) is later than in the CCV model (*t*_2_ = 0.06 ms). Correspondingly, the time when the histograms indicate a steady state diffusion process is also later in the ETV model (*t*_3_ = 10 ms) compared with the CCV model (*t*_3_ = 2 ms). One reason for the increased latency is that the half-width of the basic cell in the ETV model was *a* = 0.935 µm whereas in the CCV model it was *a* = 0.416 µm resulting in a larger ETV cellular ensemble.

#### 4.2.2. Summary of analysis of tortuosities of edge tunnel void models with *ϕ* = 0.1, 0.2 and 0.4

**Figure 7.**
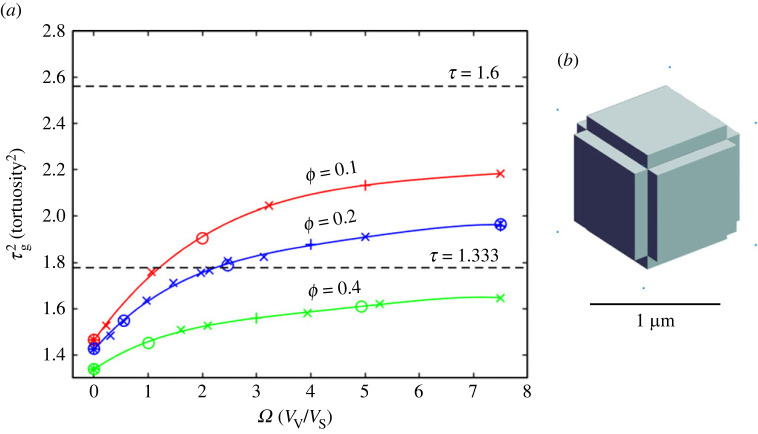
Results for ETV models with porosity in the normal range. (*a*) Porosities *ϕ* = 0.1, 0.2 and 0.4 were combined with ISS half widths *w* = 10, 20 and 40 nm. Procedures follow those for figure 4. Symbols indicate relation of data point to the chosen *w*-value; ‘o’ designates simulations with *w* = 10 nm, ‘×’ with *w* = 20 nm, ‘ + ’ with *w* = 40 nm. (*b*). ETV model that gives $\tau_{g}^{2}=1.777$ (*τ*_g_ = 1.333) for *ϕ* = 0.2, *w* = 20 nm; this is the model simulated in figure 6.

A total of 31 simulations were run with the ETV model using a similar strategy to that employed for the CCV study. The values of *b* were obtained from equation (2.17). The results of the simulations are shown in figure 7*a*. Unlike the CCV case, there is no linear dependence between $\tau_{g}^{2}$ and *Ω*. Instead, a quartic function, equation (4.3), fits the data within the range of values employed,

4.3 $$\tau_{g}^{2}=\tau_{g0}^{2}+m_{1}\Omega +m_{2}\Omega^{2}+m_{3}\Omega^{3}+m_{4}\Omega^{4}.$$

The fitting coefficients are shown in table 7.

**Table 7.** Fitting parameters for data shown in figure 7. See tables 1 and 2 for description of parameters.

*ϕ*	$\tau_{g00}^{2}$	$\tau_{g0}^{2}$	*m*_1_	*m*_2_	*m*_3_	*m*_4_	*R*^2^	*n*
0.1	1.465	1.463	0.326	−0.0610	0.00544	−0.000187	0.9996	7
0.2	1.427	1.422	0.270	−0.0668	0.00862	−0.000430	0.9985	15
0.4	1.339	1.338	0.157	−0.0443	0.00658	−0.000365	0.9993	9

Figure 7*a* shows that only the curves for *ϕ* = 0.1 and *ϕ* = 0.2 intersect the *τ*_g_ = 1.333 line ($\tau_{g}^{2}=1.777$) and none of the curves reach the *τ*_g_ = 1.6 line. Thus, this model only generates adequate geometrical tortuosity when interstitial viscosity is present. The solution to equation (4.3) for *ϕ* = 0.2, *τ*_g_ = 1.333 is *Ω* = 2.25 resulting in *a* = 0.935 µm, *b* = 0.226 µm when *w* = 20 nm (figure 7*b*).

### 4.3. Application of corner cubic void model: asleep and awake states

The results described in §4.1.2 and shown in figure 4 imply that for 0.1 ≤ *ϕ* ≤ 0.4, the relation between $\tau_{g}^{2}$ and *Ω* is linear so, in principle, it is only necessary to do one simulation with *Ω* > 0 to obtain a single value of $\tau_{g}^{2}$ because a second value can be supplied by equation (2.2), for $\tau_{g0}^{2}$ when the corner voids have shrunk to zero (*Ω* = 0). This assertion was tested by analysing data from a study that showed the ISS changing between awake and asleep states. Note that to avoid cumbersome notation, *τ*_g_ will be written as *τ* in this section and subscript ‘w’ signifies an awake state and subscript ‘s’ an asleep state. The main parameters used in §4.3 are listed in table 8.

**Table 8.** Some parameters used in §4.3.

parameter	unit	description	experimental or trial value [reference]
*ϕ*_s_		porosity in sleep state	0.234 [11]
*ϕ*_w_		porosity in awake state	0.141 [11]
*τ*_sw_		geometrical tortuosity in both states	1.55 [11] and text
*Ω*_s1_		trial *Ω* value for sleep state	4.0
*Ω*_w1_		trial *Ω* value for awake state	3.0
*τ*_s1_		geometrical tortuosity from MCell simulation using *Ω*_s1_ and *ϕ*_s_	2.031
*τ*_w1_		geometrical tortuosity from MCell simulation using *Ω*_w1_ and *ϕ*_w_	1.981
*τ*_s0_		geometrical tortuosity using *Ω* = 0 and *ϕ*_s_	1.189 (equation (2.2))
*τ*_w0_		geometrical tortuosity using *Ω* = 0 and *ϕ*_w_	1.204 (equation (2.2))

The study by Xie *et al*. [11] attracted considerable interest because it showed that in going from an awake to an asleep state, or one induced by anaesthetic, the porosity of the ISS in the mouse cortex showed a 60% increase. This finding was associated with data on the recently discovered glymphatic system to suggest that sleep facilitated the clearance of potentially neurotoxic waste products that had accumulated in the awake brain.

The ISS in the sleeping mouse cortex was characterized by *ϕ*_s_ = 0.234 ± 0.019 while in the awake state *ϕ*_w_ = 0.141 ± 0.018 [11]. Tortuosity was not significantly changed in any one state transition experiment but could vary between 1.3 and 1.8 in different experiments [11]; a constant value in the two states of *τ*_sw_ = 1.55 was chosen here. Prior studies [7] of brain porosity and tortuosity had all employed either anaesthetized *in vivo* preparations or brain slices that lack adrenergic tonic input [34]; both these preparations are functionally ‘asleep’. As before, the simulations will consider that the measured tortuosity might incorporate a viscous component equivalent to a tortuosity of 1.2. When this is factored in, the geometric tortuosity would be *τ*_sw_ = 1.292.

**Figure 8.**
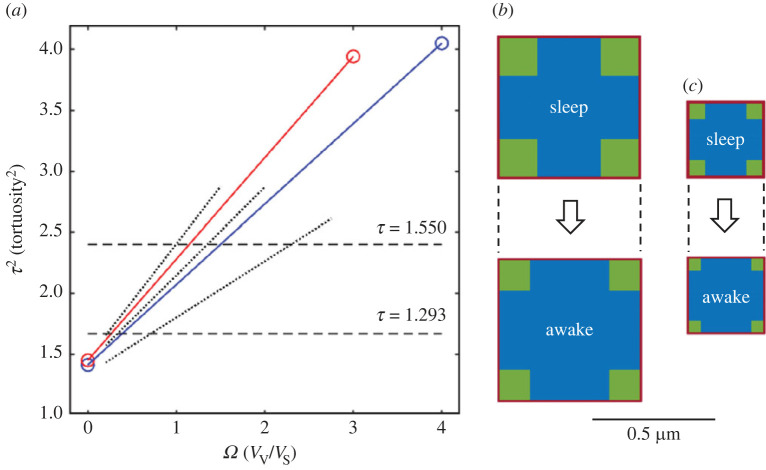
Changes in CCV model between sleep and awake states. (*a*) For sleep state, blue line connects trial *Ω*_s1_ value and simulation derived $\tau_{s1}^{2}$ to $\tau_{s0}^{2}$ at *Ω* = 0 (equation (2.2)); for awake state, red line makes similar connection. Intersection of these lines with required *τ*^2^ values (dashed horizontal lines) provide *Ω*-values that are combined with experimentally measured porosities to compute CCV geometries in sleep and awake states. Dotted lines represent partial data from figure 4 to show relation to trial lines. (*b*) Top view of CCV geometries in transition from sleep to awake state when *τ*_sw_ = 1.55. Blue is basic cell, green is void, red is atmosphere of ISS sheet around each basic cell. Dashed lines indicate that bounding cube volume remains constant between the sleep and awake states (*c*) Similar to panel (*b*) but for *τ*_sw_ = 1.295 (i.e. the case when viscosity present).

Looking at figure 4*a* it is clear that data for *ϕ*_w_ and *ϕ*_s_ should fall between the three sets of *ϕ* data (these datasets are shown as abbreviated dotted lines in figure 8*a*). Trial values for the asleep state were *ϕ*_s_ = 0.234, *Ω*_s1_ = 4.0 and for the awake state *ϕ*_w_ = 0.141, *Ω*_w1_ = 3.0. These combined with a value of *w* = 20 nm enabled the CCV geometries to be generated using equation (2.12) and equation (2.14) or equation (2.15). To check that the starting seeds did not affect the results, five MCell simulations were run, each with a different starting seed, yielding values of *τ*_s1_ = 2.013 ± 0.005 (mean ± s.d., *n* = 5) and *τ*_w1_ = 1.981 ± 0.006. The linear relation between *τ*^2^ and *Ω*, equation (4.1), was written here as

4.4 $$\tau^{2}=\tau_{0}^{2}+m\Omega ,$$

and a second pair of values were generated by employing equation (2.2), which holds when *Ω* = 0. This yielded *τ*_s0_ = 1.189 and *τ*_w0_ = 1.204.

From these data the slopes of the two lines can be determined (*m*_s_ = 0.657, *m*_w_ = 0.829) (figure 8*a*) and the four intercepts with the two *τ*_sw_ values calculated to give the *Ω* values corresponding to the experimental data (see tables 9 and 10).

**Table 9.** Sleep and awake parameters for CCV model when *τ*_sw_ = 1.55. See tables 1 and 8 for description of parameters.

Sleep: *ϕ*_s_ = 0.234, *Ω*_s_ = 1.500				Awake: *ϕ*_w_ = 0.141, *Ω*_w_ = 1.154			
*A*_s_ = 30.03, *B*_s_ = 0.5371				*A*_w_ = 43.82. *B*_w_ = 0.4324			
*w*_s_ nm	*a* µm	*b* µm	*p*_s_ µm	*w*_w_ nm	*a* µm	*b* µm	*p*_w_ µm
				10	0.4382	0.1895	0.4482
**20**	**0.6006**	**0.3226**	**0.6206**	20	0.8764	0.3789	0.8964
				40	1.7528	0.7579	1.7928
				**13.85**	**0.6068**	**0.2624**	**0.6206**

**Table 10.** Sleep and awake parameters for CCV model when *τ*_sw_ = 1.292. See tables 1 and 8 for description of parameters.

sleep: *ϕ*_s_ = 0.234, *Ω*_s_ = 0.3877				Awake: *ϕ*_w_ = 0.141, *Ω*_w_ = 0.2649			
*A*_s_ = 15.75, *B*_s_ = 0.4284				*A*_w_ = 24.89, *B*_w_ = 0.3215			
*w*_s_ nm	*a* µm	*b* µm	*p*_s_ µm	*w*_w_ nm	*a* µm	*b* µm	*p*_w_ µm
				10	0.2489	0.0800	0.2589
**20**	**0.3150**	**0.1350**	**0.3350**	20	0.4977	0.1600	0.5177
				40	0.9955	0.3201	1.0355
				**12.94**	**0.3221**	**0.1035**	**0.3350**

In this section the interest lies in how the geometry changes in going from the sleeping to the awake state; the sleeping state is taken as the reference because most prior work is in this state. We know that the porosity decreases but cannot say which parts of the ISS change. Looking at table 9 (table 10 will follow a similar logic) and taking the sleep geometry for *w* = 20 nm as a reference, it is seen that, if there is no change in *w* then the void width, *b*, will have to *increase* even though overall porosity *decreases*. To resolve this paradox, the half-width of the basic cell, *a*, must increase (i.e. the cell swells) and the bounding cube (half width *p*) also must increase so the tissue swells because *p*_s_ < *p*_w_. By contrast, if *w* is reduced to 10 nm in the waking state, then *b* and cell size decrease leading to a reduction in *p* and tissue shrinkage. In the unlikely event that *w* increases to 40 nm then there would have to be a dramatic enlargement of both cell and void.

This analysis raises the question of what happens when there is no change in the bounding cube volume in going from sleep to the awake state i.e. *p*_s_ = *p*_w_. Simple manipulation based on equation (2.12) indicates that

4.5 $$w_{w}=\frac{\;p_{s}}{\left(1+A_{w}\right)}.$$

This leads to the bottom right row in table 9. Now both *w* and *b* decrease and *a* increases slightly to compensate for the reduction in *w*. This is the most plausible scenario because it would be undesirable for the brain to change volume between asleep and awake states. These same arguments are replicated if *τ*_sw_ = 1.292, i.e. in the case of a viscous ISS, although the dimensions involved are different (table 10).

## 5. Discussion

Brain tissue appears vastly complex when viewed with an electron microscope. More than 60 years of study of the diffusion of small molecules shows, however, that the interstitial space (ISS) of the brain i.e. the labyrinth of narrow channels between cells, strongly resembles the pore space of an unconsolidated porous medium. Indeed, these studies of diffusion have most frequently described the results in terms of porosity (often called volume fraction by neuroscientists) and tortuosity; both parameters well known to the porous media community. A challenge in brain studies has been to account for the measured tortuosity in terms of the cytoarchitecture of brain tissue. Both porosity and tortuosity take a narrow range of values even when measured in different brain regions and different species [7]. Here we study two simple models of brain tissue and show that a combination of ‘sheets’ and ‘voids’ is sufficient to account for the experimental data. By sheets is meant narrow, uniform, gaps between adjacent brain cell membranes and by voids is meant local enlargements of the ISS in the form either of cavities that are only connected via the sheets, or voids that take the form of tunnels, tube-like expansions, that are wider than the sheets and interconnected in a lattice.

A possible complication in interpreting the diffusion experiments in terms of pure geometry, is that the ISS may have an intrinsic viscosity that makes a non-geometrical contribution to the measured tortuosity. Such viscosity might represent a non-specfic interaction with the extracellular matrix; further characterization of the matrix structure and distribution could enable future Monte Carlo simulations to represent the interaction in detail. Here results from the only attempt to measure ISS viscosity [22] have been used to construct corner cubic void (CCV) and edge tunnel void (ETV) models taking account of viscosity. The conclusion is that the putative viscosity affects some quantitative results, such as basic cell size, but it does not remove the necessity for sheets and voids to explain the diffusion data.

Prior work [28–30] has shown that representing brain cells as packed ensembles of cubes and other convex cell shapes, separated only by uniform sheets, all produce a similar tortuosity, but the value is always lower than that measured in brain tissue. This discovery prompted the introduction of dead space microdomains into models of the ISS while still retaining the simple cubic cell; such dead spaces increase geometrical tortuosity. In this study, brain tissue is represented by ensembles of basic cellular structures consisting of a CCV or ETV cell and its enveloping atmosphere of sheets. The sizes of the sheets and voids have been adjusted to give a required porosity and tortuosity measured by Monte Carlo simulations of diffusion with an ensemble of model cells and a population of molecules released from a point source. The evolutions in time of the tortuosity shown in figures 3 and 6 are typical for the selected parameter values. For all sets of values the calculated tortuosity rises from a value of 1 to a steady state value over a variable period that is measured in milliseconds and then remains constant. The shape of the curve varies with the parameters, as does the time to steady state and it is only when the steady state is attained that the tortuosity has a well-defined meaning based on a Gaussian distribution of molecules.

A ‘lake and gaps’ model that resembled the CCV model was used by Jin *et al*. [35] to try to account for brain diffusion data they obtained with several fluorescent molecules using microfiberoptic photobleaching. The lake model failed to replicate the experimental results. Based on the present work it appears that Jin *et al*. used a basic cell that was at least five times too large. In addition, an attempt to reproduce some of Jin *et al*.'s model data, using the CCV model described here, revealed apparent errors in several published values. The errors may have arisen from the unusual Monte Carlo algorithm employed by those investigators.

Several new findings emerge from the present study. Perhaps the most interesting is that for the CCV model with porosity *ϕ* in the anatomically plausible range (*ϕ* ≤ 0.4), $\tau_{g}^{2}$ (*τ*_g_ is geometrical tortuosity) is linearly related to *Ω* (*Ω* is void volume/sheet volume). Moreover, within this linear domain, large tortuosities are possible and the experimentally obtained values are easily reached. The typical basic cubic cells that emerge in this study have widths (2*a*) of 0.5–2.6 µm (table 4). This size is consistent with the dimensions of dendrites, unmyelinated axons, and glial processes in the neuropil of the brain (figure 1). Cell bodies are considerably larger, but they are sparsely distributed in the neuropil. The voids explored in this study are spaced apart at the width of the bounding cell, i.e. 2*p* µm, and so one might regard this work as a study of voids with this spacing. The typical void width (2*b*) is 0.3–1 µm (table 4) which is also consistent with microscopy (figure 1).

Taking the CCV model into the anatomically non-realistic realm (0.6 ≤ *ϕ* ≤ 0.9), there is no longer a linear relation between $\tau_{g}^{2}$ and *Ω* and the basic cells attain a striking lamella geometry for extreme porosity. Surprisingly, even at *ϕ* = 0.9, experimentally realistic tortuosities are obtainable. This definitively demonstrates that *ϕ* and *τ*_g_ need not be related. This is important because many studies in the porous media literature attempt to derive relations between these two parameters; such relations may be valid for specific models but cannot be generalized.

Diffusion in the ETV model evolves in a similar way to the CCV but the linear relationship between $\tau_{g}^{2}$ and *Ω* is absent and the model can only generate slightly greater tortuosities than an ensemble of simple cubes without voids. The ETV model cannot produce *τ*_g_ = 1.6; the model can only generate *τ*_g_ = 1.333, the value when interstitial viscosity is present. The occurrence of the wider voids in the tunnels compared with sheets should increase tortuosity but this is apparently counteracted by the increased connectivity of the tunnels. Thus, the ‘sheet and tunnel’ model, at least in the form presented here, is not as versatile or plausible as the CCV model. Note that the existence of voids, as employed in the CCV model, is based on considerable evidence, tunnels are less characterized because of the need for three-dimensional electron microscopy reconstruction.

While the ISS has many characteristics of a porous medium, one property that may be less common in the physics and engineering worlds is the ability of brain tissue to change its dimensions under some conditions. Recent work has shown that the ISS changes porosity in going between asleep and awake brain states and this intriguing fact lends itself to an application of the CCV model. Imposing the condition that the brain neither swells nor shrinks in transitioning between sleep and wakefulness, results in unique changes in the sheet width, void size and basic cell volume while retaining a constant bounding cell size.

The CCV model is simple but versatile and tables 3–5 provide data for construction of models that can be used to predict diffusion and other transport modalities in the ISS, such as advection or electrical conductivity. If other models are required, for example to mimic ISS conditions during brain ischaemia [7], the methodology in §4.3 should be applicable.

## Data Availability

All the data for study are included in the figures and tables. Typical MCell scripts for the Monte Carlo simulations and usage instructions are included as a zipped electronic supplementary material file [36].
